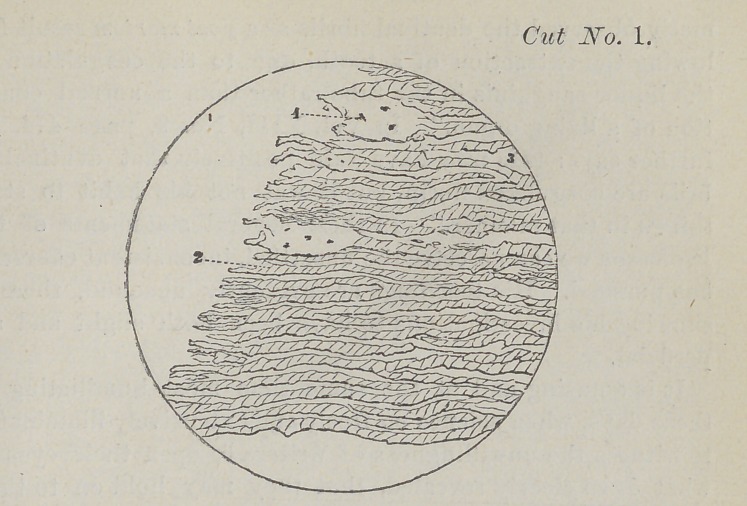# Is the Dentine Tubular?

**Published:** 1872-09

**Authors:** Geo. B. Harriman

**Affiliations:** Boston, Mass.


### Dental Register.

Vol. XXVI.] SEPTEMBER, 1872. No. 9.]

### COMMUNICATIONS.

### Is the Dentine Tubular?

PROF. GEO. B. HARRIMAN, BOSTON, MASS.

The attentive reader of my preceding articles, relating to the constituent parts of the teeth and the manner of their formation, has not discovered any allusions to "prisms," or "pillars," or "tubes," or "canals," or "hollow columns," "tubuli or hard pipes, etc," as inhering to any appendage of a tooth. These terms are common in treatises on the subject, and are used by professors in dental colleges when addressing their pupils, and in their communications to the press. These terms are frequent in the writings of Kolliker and other authors; and it may be deemed an act of arrogance to ignore such terms as having no foundation in any development of the teeth, or in any part of the human body.

I have been constrained to take this position after much study, investigation and experiment. I am unwilling to employ these terms, for the reason that they do not announce what exists, and that they express something which the most rigid examination will fail to discover. I am confident I can demonstrate to any competent and candid mind that these so-called tubuli are real cellular elements, as much so as are the cells in cartilage, connective tissues, or the spinal column. Where is the learned histologist, who either directly or indirectly admits that human life anywhere is nourished, sustained, and advanced to maturity in any other manner than through and by the action of the cell? He is hardly to be found at the present day. Schwaum, Reichert, Huxley, Remark, Virchow and others, advocate clearly the cell theory in every other tissue of the animal and vegetable formation and growth. The spermatosoa is a cell from which the proliferation of the entire body is produced. Take the skin, for instance, of a full-grown individual—it becomes irritated by reason of continued friction, and the irritation thickens; and if the proliferation is very active it may lead to the production of quite large tumor-like formations.

The great point in the application of histology is to obtain the recognition of the *fact* that the cell is really the morphological element in which there is any manifestation of life. It is impossible to transfer the real seat of action to any point beyond the cell. In the muscles, in cartilage, in connective tissue, and in nerves we discover the same organization and proliferation. In all the tissues we detect a nucleus, and at times two nuclei, with a division taking place in the structure of exisiting cells.

This law obtains in the growth of the teeth. At first there can only be discerned this cellular element; cells with nucleus and in some instances nuclei, and a division taken place, the nuclei being the starting point of the new cells. As the calcareous salts continue to be desposited intersticially, the cells arrange in lines forming fibres till there is the development of the entire tooth. If we take a thin shaving from a fully developed tooth, and treat it to acetic acid, watching it with care, we shall ascertain that it is composed of cells, closely impacted, edge to edge; and when the calcareous substance is all dissolved, at the anastomosis of these cells, and fibres can be seen in very many instances the nucleus of the cell.

*Cut No.* 1.

This cut represents the cells and fibres of a central incisor tooth. Figure one points to the nucleus after the application of acetic acid. Figure two shows the cells closely impacted. Figure three represents the lime salts. I am certain that the dichotomous or forked-like bifurcations sometimes presented in a section of a tooth under the microscope, are caused by the union of cells. Prof. McQuillen (*Dental Cosmos*, Vol. VII., No. 9, page 454), has a cut of a transverse section, showing what he is pleased to call the intertubular tissue and dentinal tubuli. His dentinal tubuli quite accurately describes a cell with a nucleus, but entirely fails to bring forward and show a hollow tube, which it was his design and desire to do. On the page above quoted he says: "Tubuli originate by open mouths on the walls of the pulp cavity, and proceed in a slightly wavy and radiated manner through every portion of the dentine to its periphery, where they generally terminate; though in some instances extending beyond and penetrating the enamel or cementum, in the latter of which they communicate with the lacunæ through the canaliculi." In a cut in the volume and number above quoted, page 455, he endeavors to illustrate this position. In Vol. XIII, No, 1, page 37, the Professor remarks: "A section from a recently extracted tooth evidently consists of hard dentine and a soft, solid subtance." In Vol. IX, No. 5, page 231, he says: "I have formerly observed the dentinal fibrils as a *post mortem* result following the extraction of a tooth, due to the coagulation of the liquor sanguinis in the tube rather than a normal condition of a living organ." In Vol. XIII, No. 9, page 474, he further says: "He may have said positively that dentinal fibrils are coagulated fibrin, but it was not his habit to state things in that manner." In these several statements of the Professor we can perceive the unsettled, inconsistent course he has pursued. His teachings are evidently unsound, thereby showing his investigations to have been both slight and superficial.

It is amusing and at the same time most humiliating in these days, when science so freely and extensively illuminates, to witness the unwillingness of writers to open their eyes to what is so clearly revealed, that they may hold on to their old opinions however absurd or unsound, and untruthful. The day has passed for either a professor or teacher to be accounted great or of any account for being wrapped in the mantles of the learned of antiquity. Those mantles were cut and made by meagre light, and poorly compare with what now are worn by the great and learned. The general intelligence of the people at the present period, and the general desire and practice of professional men to admit neither principles nor terms which are unsound or untruthful. This feeling is so strong, that any member of either of the professions who rebels, and is in the leading strings of antiquity, will find himself without renown and professional success, and reward.

Now when it is so easy to explode such terms as "tubuli," it is mortifying to have men making themselves ridiculous by saying that "tubuli originates by open mouths," This definition is accompanied with a cut which is altogether pictorial, and descriptive of nothing which is real. After most laborious and long search among almost endless experiments, I am unable to discover any formation "tubuli or lacunæ," unless cells and fibres are such. It appears to me Prof. McQuillen himself disproves his own theory when he says, "the wavy character of the tubuli above appears to consist of two or three large or primary curvatures, which follow closely upon each other, and are estimated by Retrius as numerous as two thousand in a single line." If these *curvatures* are called *fibres*, microscopic examination will approve. If they are called tubuli, etc., the dark ages can alone give the reason. We are to burrow under old authorities, cleaving to what can stand and discarding what is unsound and disproved by present scientific developments.

## Figures and Tables

**Cut No. 1. f1:**